# MicroRNA (miR) 125b regulates cell growth and invasion in pediatric low grade glioma

**DOI:** 10.1038/s41598-018-30942-4

**Published:** 2018-08-21

**Authors:** Ming Yuan, Ana Cristina A. L. Da Silva, Antje Arnold, Laurence Okeke, Heather Ames, Lina S. Correa-Cerro, M. Adelita Vizcaino, Cheng-Ying Ho, Charles G. Eberhart, Fausto J. Rodriguez

**Affiliations:** 1 Division of Neuropathology, Johns Hopkins University School of Medicine, Baltimore, MD USA; 2 Federal University of Uberlandia, Uberlandia, Brazil; 3 Department of Cellular and Tissue Biology, Faculty of Medicine, UNAM, Mexico City, Mexico; 4 Department of Pathology, University of Maryland, Baltimore, MD USA; 5 Sydney Kimmel Comprehensive Cancer Center, Johns Hopkins University School of Medicine, Baltimore, MD USA

## Abstract

Members of the miR-125 family are strongly expressed in several tissues, particularly brain, but may be dysregulated in cancer including adult and pediatric glioma. In this study, miR-125 members were downregulated in pilocytic astrocytoma (PA) as a group compared to non-neoplastic brain in the Agilent platform. In the Nanostring platform, miR-125 members were downregulated primarily in pleomorphic xanthoastrocytomas and gangliogliomas. Using CISH for miR-125b, highest levels of expression were present in grade II tumors (11/33, 33% grade II tumors with 3+ expression compared to 3/70, 4% grade I tumors) (p < 0.001). When focusing on the two histologic subgroups with the largest number of samples, PA and diffuse astrocytoma (DA), the highest expression levels were present in DA, in comparison with the PA group (p = 0.01). Overexpression of miR-125b in pediatric low grade glioma (PLGG) derived cell lines (Res186, Res259, and BT66) resulted in decreased growth and invasion, as well as apoptosis. Additionally, miR-125b overexpression in BT66 resulted in senescence. These findings suggest that miR-125 is frequently underexpressed in PLGG, and overexpression results in a decrease in cell growth and induction of apoptosis, findings that deserve further investigation given its potential as a novel therapeutic strategy for PLGG.

## Introduction

Pediatric low-grade glioma (PLGG) represents an heterogeneous group of primary CNS neoplasms characterized by slow growth. Glioneuronal tumors are somewhat related given that a low-grade glioma component is frequent and they are also characterized by similar demographics. The prognosis for these tumors is generally good, but complete surgical resection may not be possible in specific locations, therefore they have a potential for morbidity related to mass effect.

High resolution platforms have uncovered recurrent alterations in these tumors, i.e. *BRAF-KIAA1549* fusions as the most frequent recurrent alteration in pilocytic astrocytoma (PA)^1–5^, which represents the most frequent subtype of PLGG. *BRAF-KIAA1549* fusions lead to mitogen-activated protein kinase (MAPK)^2^. Interestingly, comprehensive sequencing studies reported genetic alterations in MAPK components in essentially all (100%) of PA^6^. PLGG also have heterogeneous histologies, including neoplasms with infiltrating astrocytic or oligodendroglial components, which may have a variety of alterations in alternative drivers (*FGFR1, MYB, MYBL1*)^7,8^.

The role of non-coding RNAs, particularly microRNAs, is an active field of study in normal physiology and a variety of disorders, including cancer. MicroRNA upregulation may target tumor suppressors, while downregulation of microRNAs may result in increased levels of oncoproteins. In the field of glioma, alterations in the expression of numerous microRNAs have been described, and they may play a role in every known process associated with glioma biology (reviewed in^9^). PLGG in particular are an attractive subject for microRNA profiling, given their relative lack of genomic instability in contrast to high grade tumors^10^. Most microRNA profiling studies in gliomas have been performed in adults, particularly high grade and diffuse subsets such as glioblastoma, where they appear to mediate a variety of important tumor properties (reviewed in^9^). Conversely, studies of microRNA expression in circumscribed and low grade pediatric gliomas are relatively scant. When focusing on pediatric glioblastoma, and comparing expression levels with non-neoplastic brains, studies have documented relative under-expression of miR-1224, miR-204, miR-874, miR-1296, miR-889, miR-495, miR-34c, miR-10b, miR-125a, miR-10a, while miR-617, miR-200a, miR769-3, miR-584, and miR-527 are overexpressed^11,12^.

When focusing on PLGG, miR-21 and miR-34 overexpression have been consistently documented in PA^11–14^. Several of the predicted targets of these microRNAs involve components responsible for regulation of the MAPK signaling pathway, which is universally activated, predominantly through a *BRAF-KIAA1549* fusion gene product^6^. Our prior studies and other investigators have reported additionally relatively underexpressed microRNAs in PA compared to normal brain, including miR-124 and miR-129^11,12,14,15^. Functional validation of these microRNAs in PLGG has limitations, primarily given the relative lack of *in vitro* and *in vivo* models of this disease. Our group has recently documented underexpression of miR-487b in PA, and *in vitro* experiments demonstrated downregulation of putative targets/stem cell markers PROM1 and nestin and decreased colony formation in soft agar when miR-487b is overexpressed^13^. Comprehensive studies of microRNA regulation in glioneuronal tumors are even more scant, with rare reports of alterations in inflammation-associated microRNAs in ganglioglioma^16,17^.

miR-125b is the human homologue of one of the first microRNAs discovered, i.e. lin-4 in *C. elegans*^18,19^, and is evolutionarily conserved. The miR-125 family encompasses three homologues hsa-miR-125a, hsa-miR-125b-1 and hsa-miR-125-2, and have various roles in physiology and disease (reviewed in^20^). The list of putative targets is wide and contains transcripts involved in proliferation, metastasis, differentiation and immune regulation. In solid tumors, a tumor suppressor role for miR-125 in cancers of lung, ovary, breast, bladder, bone and skin has been documented. Wang *et al*. reported that miR-125a is regulated by EGFR signaling and leads to decreased invasion and migration in lung cancer cells^21^. In breast cancer, miR-125 is downregulated and affects ERBB2 and ERBB3 signaling and invasion properties^22^, while ectopic regulation of miR-125b regulates proliferation in hepatocellular carcinoma^23^. Additionally, miR-125b may play a role in apoptosis. For example, Morelli E. *et al*. demonstrated that miR-125b overexpression in multiple myeloma cells leads to apoptosis, likely mediated through IRF4 downregulation^24^.

It must be noted that miR-125b has been also found to act as an oncogene in certain contexts, including cancers of endometrium, breast and prostate^20^. When focusing in glioma, there have been reports of miR-125b acting as an oncogene in glioblastoma, through targeting of *TP53*^25^, and connexin43^26^. Additional studies have reported that miR-125b regulates the proliferation of glioma stem cells through E2F2 regulation^27^. The role of miR-125b in low grade glioma has not been thoroughly studied to our knowledge. Of interest, Nyholm *et al*. demonstrated that miR-125b leads to cellular senescence in melanoma, specifically G0/G1 cell cycle arrest, morphologic changes, beta galactosidase staining and increased levels of p53, p21 and p27.^28^. In the same study, there was not a significant increase in apoptosis. PLGG shares with melanoma a high prevalence of activating mutations in *BRAF*. Furthermore, overexpression of active BRAF leads to oncogene induced senescence in PLGG^29,30^, which may be one of the reasons that this disease is difficult to model in the laboratory. To our knowledge, the role of miR-125b in PLGG, and specifically senescence has not been studied. In this study, we searched for putative microRNAs associated with senescence in PLGG, and uncovered miR-125b as downregulated in low grade gliomas and glioneuronal tumors and associated with tumor suppressive functions. We therefore evaluated the expression of miR-125b in PLGG and similar BRAF activated tumors in adults and studied the functional effects of miR-125b overexpression in PLGG cells lines.

## Results

### miR-125b is downregulated in low grade glioma and glioneuronal tumors in microRNA expression datasets

To identify biologically relevant microRNAs for PLGG we performed a literature search of altered microRNAs in the context of senescence^31,32^ and dysregulated in tumors associated with *BRAF* alterations^28,33^. We searched our previously published^13,14^ and unpublished microRNA data, and miR-125b was consistently downregulated in low grade glioma/glioneuronal tumors when compared to normal brain.

**Figure 1:**
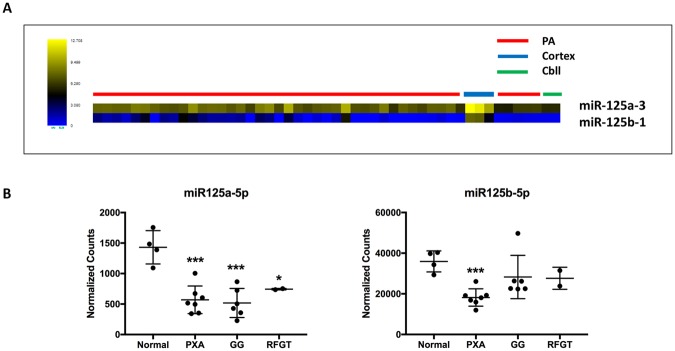
miR-125 is downregulated in pilocytic astrocytoma (PA) and other low grade gliomas/glioneuromal tumor compared with non-neoplastic brain. MicroRNA expression data obtained through Agilent arrays demonstrated lower miR-125 expression levels in PA (n = 43) compared to non-neoplastic brain, particularly cerebral cortex (n = 3) (cbll = cerebellum n = 2). Each data point (heatmap square) represents an individual sample. (**A**) microRNA expression using a nanostring platform demonstrated miR-125 underexpression particularly in pleomorphic xanthoastrocytoma (PXA) (n = 7), ganglioglioma (GG) (n = 6) and rosette forming glioneuronal tumor (RFGT) (n = 2). Each point represent an individual sample. Only these 3 subgroups that demonstrated significant differences between tumor and normal brain are illustrated. Normalized counts were obtained using nSolver software (NanoString) on Raw data filtered for minimum expression threshold, and log2 transformed before analysis as previously reported^13^ (**B**).

First, we looked at a dataset of 43 PA and 5 non-neoplastic brain samples profiled with the Human miRNA Microarray V3 Agilent platform using frozen surgical tissues^14^ which tested for miR-125a-3p, miR-125a-5p, miR-125b, miR-125b-1 *and miR-125b-2*. In this experiment, only miR-125a-3p and miR-125b-1* were relatively underexpressed in tumors 3 fold and 6 fold respectively compared to non-neoplastic brain. The differences were most pronounced in cerebral cortex, and less so in pediatric/fetal cerebellum (Fig. 1A). In a second dataset obtained from formalin-fixed paraffin-embedded tissues, a variety of low grade glial and glioneuronal tumor pathologic subtypes (n = 41) and 4 brain samples were profiled with the Nanostring platform^13^, which includes miR-125b-5p, miR-125a-5p, miR-125a-3p, In this experiment, hsa-miR-125a-5p and miR-125b-5p were underexpressed 2.5 and 2 fold respectively in pleomorphic xanthoastrocytomas (n = 7), and miR-125a-5p was underexpresed 2.8 and 2.4 fold in gangliogliomas (n = 6) and rosette forming glioneuronal tumors (n = 2) respectively compared to brain (Fig. 1B). There were no significant expression differences in the other tumor types or miR-125 family members (not shown).

**Figure 2:**
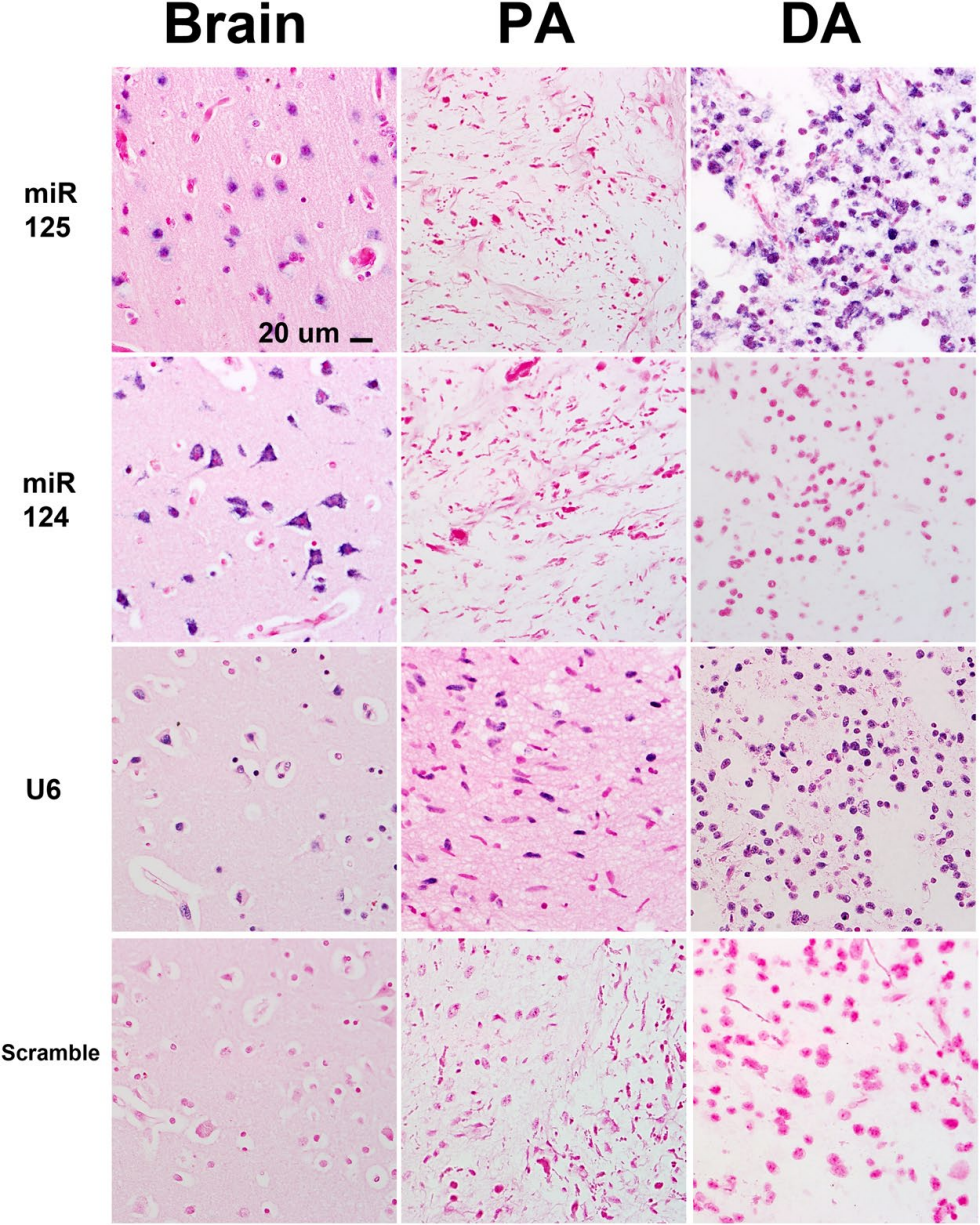
Differential miR-125b expression in low grade glioma subsets. MiR-125b expression detected by *in situ* hybridization was lower in pilocytic astrocytoma (PA) compared to diffuse astrocytomas (DA) and non-neoplastic brain (“brain”). Conversely, miR-124, a ubiquitous microRNA expressed in neurons was underexpressed in all gliomas tested. U6 was expressed in all tissues, while the scramble (negative) control was negative. Figures are representative of each group tested and all are taken with a 40X objective. The scale bar on the upper left figure applies to all figures in the panel.

**Figure 3:**
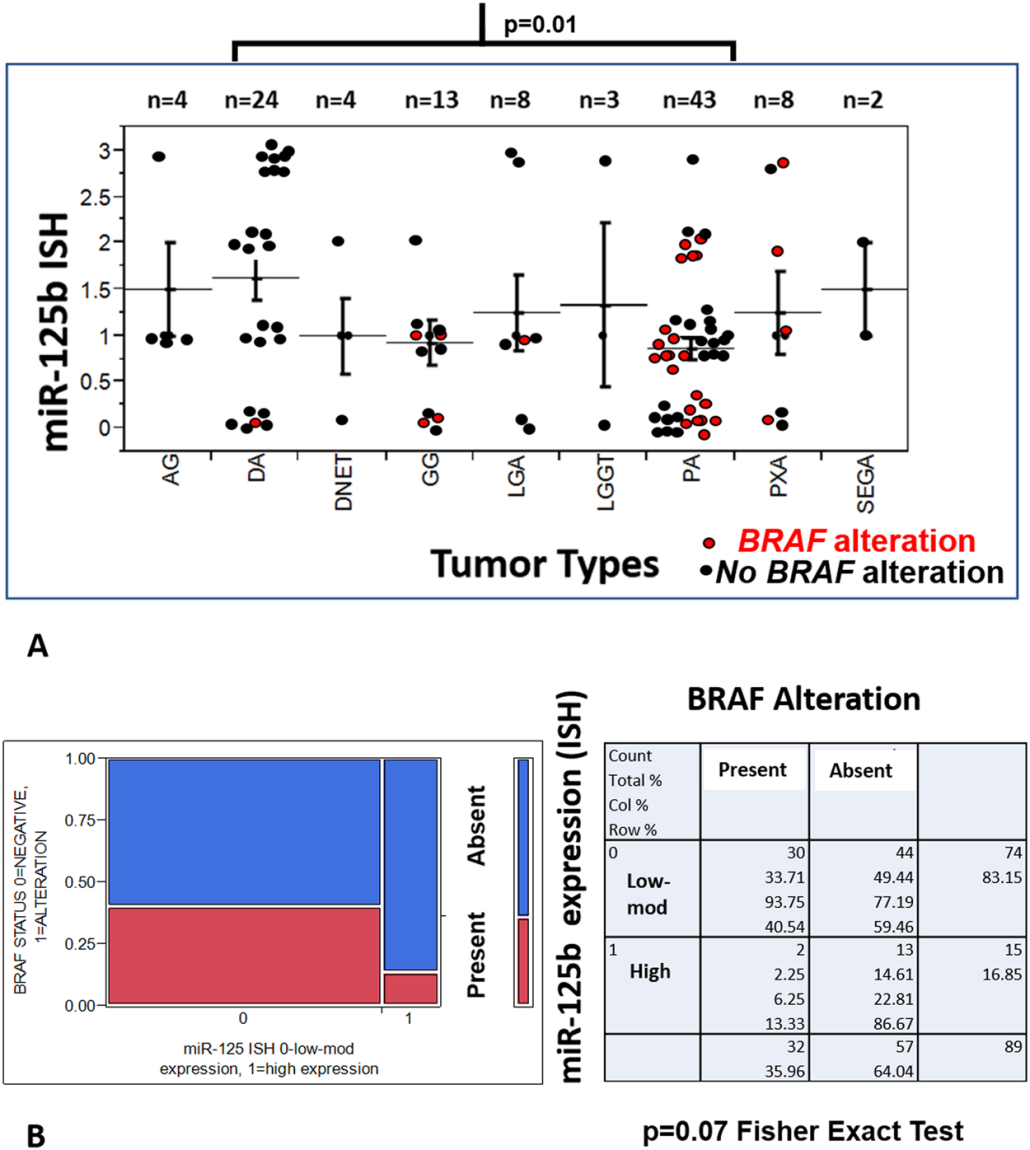
miR-125b expression is downregulated in pilocytic astrocytoma, and other circumscribed gliomas compared to diffuse astrocytoma. *In situ* hybridization studies using probes targetting miR-125b demonstrated variable levels in low grade gliomas and glioneuronal tumors, but levels were highest in diffuse astrocytoma (DA) and lowest in pilocytic astrocytoma (PA) (p = 0.01) (Wilcoxon Rank Sum Test). Strength of ISH hybridization was scored using a 4 tiered scale semiquantitative scale, ranging from 3 (strong, similar to brain), 2 (moderate), 1 (weak), 0 (negative) (y-axis). Each dot represents an individual tumor, and *BRAF* mutated tumors are in red color (AG = angiocentric glioma, DNET = dysembryoplastic neuroepithelial tumor, GG = ganglioglioma, LGA = low grade astrocytoma, LGGT = low grade glioneuronal tumor, PXA = pleomorphic xanthoastrocytoma, SEGA = subependymal giant cell astrocytoma) (**A**). There was a non-significant trend for high miR-125b expression in tumors lacking *BRAF* alterations (*BRAF* duplications or BRAF p.V600E) as demonstrated by the two tailed Fisher exact test and contingency table (**B**).

Next, we studied miR-125b expression in 125 low grade tumors of 9 different histologic subtypes using CISH, with statistical comparisons limited to the largest groups. Although absent and low reactivity were identified in both grade I and grade II tumors, highest levels of expression were present in grade II tumors (11/33, 33% grade II tumors with 3+ expression compared to 3/70, 4% grade I tumors) (p < 0.001) (Figs 2 and 3A). miR-125b expression was also preserved in non-neoplastic cells (neurons) within grade I and grade II tumors. There was a non-significant trend for high miR-125b expression in tumors lacking *BRAF* alterations (high expression in 13/57, 23% tumors lacking BRAF alterations vs 2/32, 6% tumors with *BRAF* duplication or *BRAF* p.V600E) (p = 0.07, 2-tail Fisher exact test) (Fig. 3B). When focusing on the two histologic subgroups with the largest number of samples (PA, DA), the highest expression levels were present in DA (n = 24), in comparison with the PA (n = 43) group (p = 0.01) (Wilcoxon Rank Sum Test).

### miR-125b expression is frequent in non-neoplastic brain and lower in neural stem cell compartments

We next assessed miR-125b expression levels in non-neoplastic human tissues. First we looked at a published comprehensive dataset of microRNA expression in 114 non-neoplastic and cancer tissues and cells lines (“the human microRNome”) obtained through small RNA-seq reads^34^. Increased expression levels, as represented in reads per million (RPM), were overrepresented in central nervous system (CNS) derived tissues and cells, particularly prefrontal cortex (22,005 RPM) and retinal pigment epithelium (RPE) (67150 RPM). Increased expression was also noted in skin (58488 RPM), cervix (30022 RPM), valve interstitial cell (23430 RPM) and osteoblasts (21736 RPM) (Supplementary Table 3).

To better define the range of miR-125b expression in mature brain and development we studied various brain regions from 6 patients with non-neoplastic processes using CISH which was uniformly expressed in all samples. (Supplementary Fig. 1 shows representative images of expression in mature non-neoplastic brain. In mature brain, strong miR-125b expression (3+) was evident in neurons of the neocortex (Supplementary Fig. 1A), spinal cord, dentate nucleus, cerebellum (Purkinje cells, dentate nucleus, internal granular layer), reactive astrocytes, arachnoidal cells and ependymal cells. miR-125b was weak to absent in white matter (Supplementary Fig. 1B).

**Figure 4:**
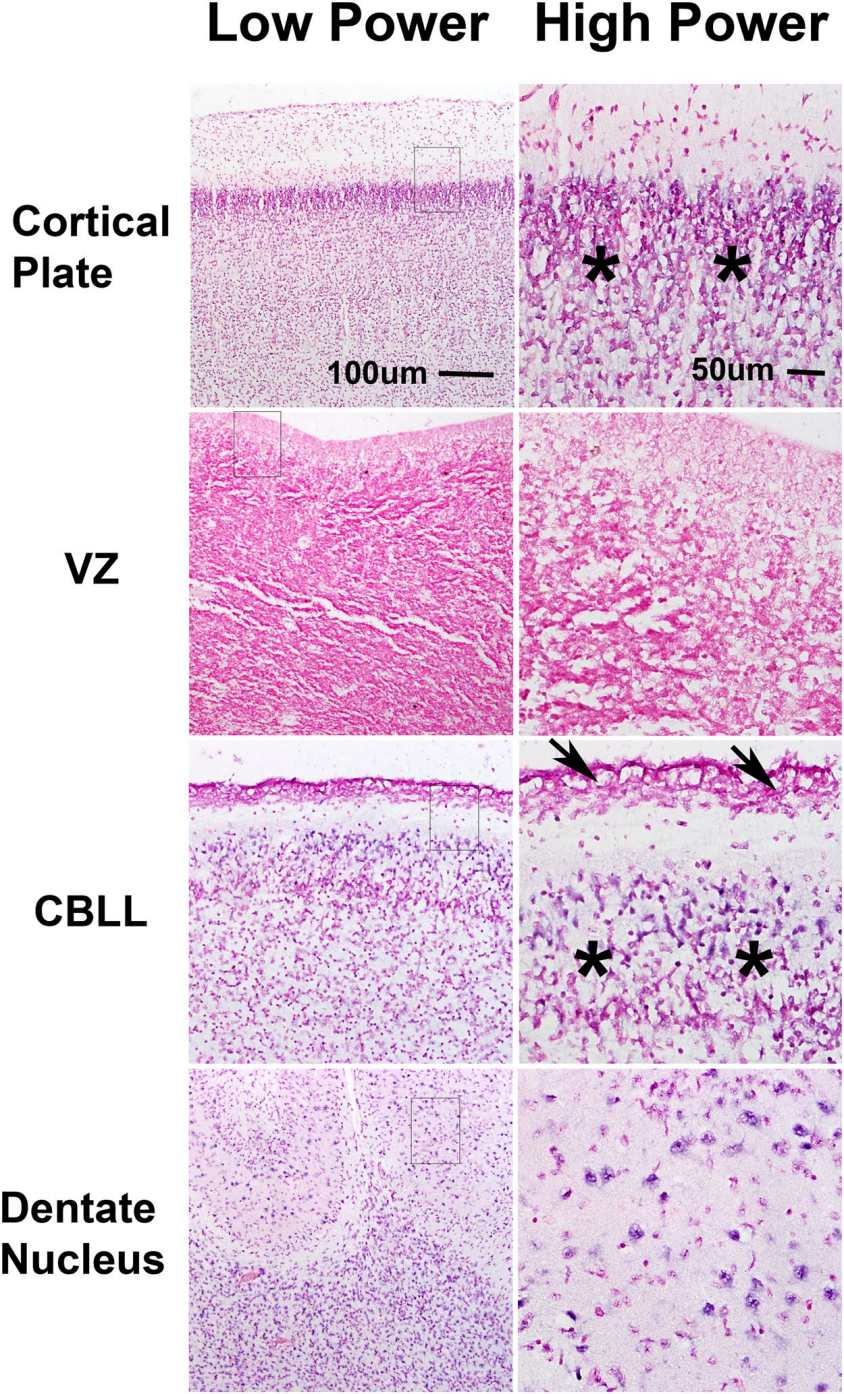
miR-125 expression varies in different fetal brain cell compartments. miR-125b expression studied in a 20 week-old fetal brain. miR-125b expression was preserved in maturing CNS cellular elements, including the cortical plate and the internal granular cell layer of the cerebellum (CBLL) (asterisks), but lost in stem cell niches, such as the ventricular zone (VZ) and the external granular cell layer (arrows). miR-125b expression was also preserved in neurons of the dentate nucleus and developing white matter. Scale bars for low power and high power figures on top apply to all correspondng low and high power figures below. Low power views are taking using a 10X objective while high power views are taken with a 20X objective.

In both fetal samples examined, miR-125b was expressed in the cortical plate, cerebellar internal granular cell layer, and hippocampus (dentate gyrus and cortex). Expression was absent in the external granular cell layer and ventricular zone (representative images of expression in both samples illustrated in Fig. 4). These findings suggest that miR-125b expression is a feature of mature neuronal and astrocytic cells, but is decreased in fetal neural stem cell compartments.

### miR-125b overexpression halts cell growth in pediatric low grade (PLGG)-derived cell lines

**Figure 5:**
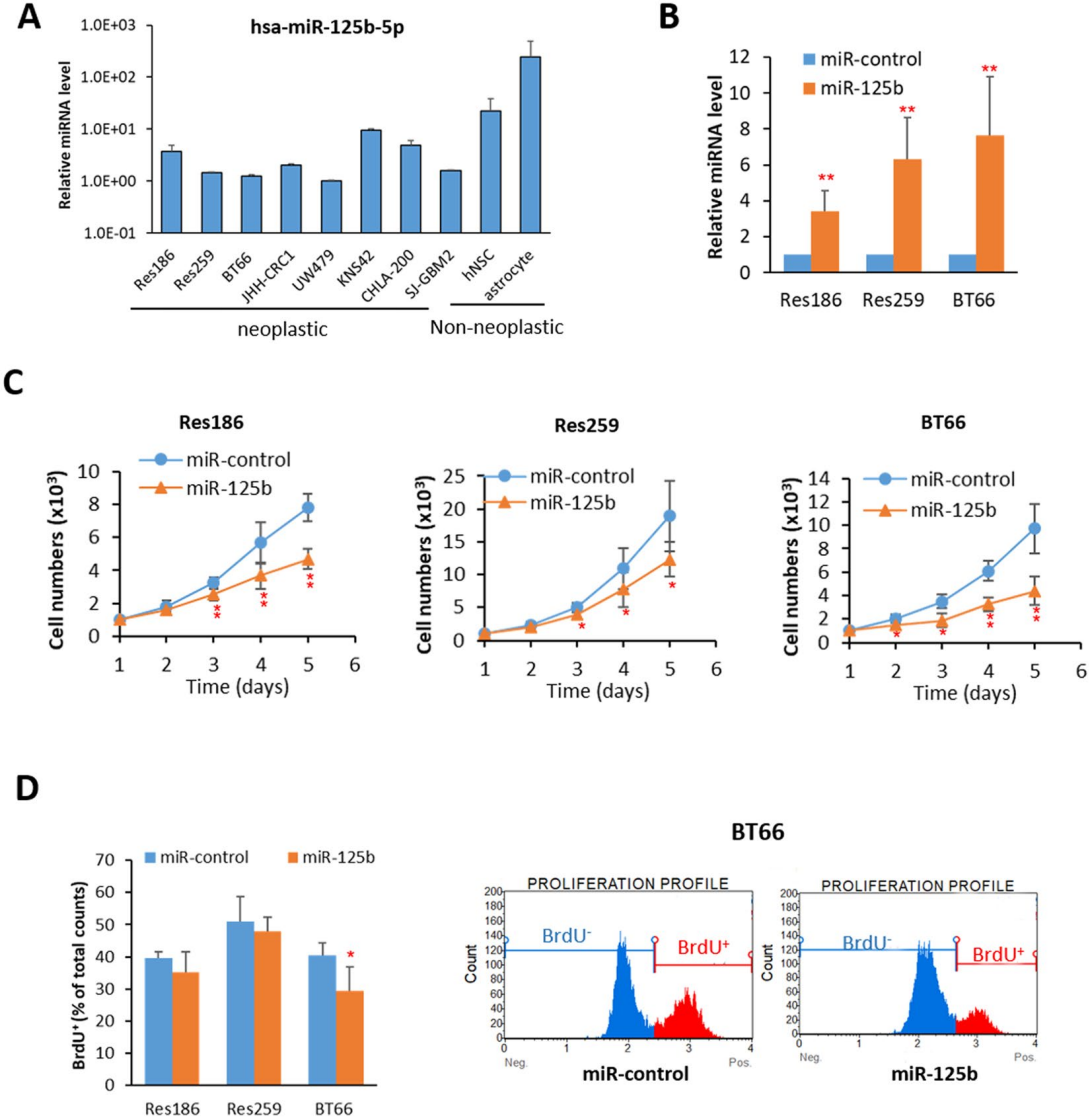
Overexpression of miR-125b decreases cell growth. (**A**) qRT-PCR of miR-125b-5p expression in pediatric cell lines. (**B**) Overexpression of miR-125b in Res186, Res259 and BT66 (*BRAF* fusion+) cells. All data were normalized with miR-control infected cells. (**p < 0.01 compare to miR-control). (**C**) Cell growth curve of Res186, Res259 and BT66 cells infected with miR-control and miR-125b. (*p < 0.05 and **p < 0.01 compare to miR-control). (**D**) Quantification of BrdU incorporation is at left and BT66 analyzed data of flow cytometer is at right (*p < 0.05 compare to miR-control). Results represent the average of at least three biological replicates and data were analyzed with a two-tailed Student’s t-test.

Next we studied various glioma cell lines for miR-125b-5p expression using qRT-PCR. The expression of miR-125b-5p was variable across the glioma cell lines but uniformly lower than non-neoplastic cell cultures, including mature astrocytes and human neural stem cells (hNSC) (Fig. 5A). In contrast, expression of miR-125b-1-3p and miR-125b-2-3p was similar to non-neoplastic cells, with the exception of decreases in miR-125b-1-3p in UW479 and miR-125b-2-3p in BT66 and UW479 (Supplementary Fig. 2). Given our prior observations demonstrating decreased levels of miR-125 family members in PLGG, we selected Res186, Res259 (both lacking *BRAF* alterations) and BT66 (which carries a *BRAF-KIAA1549* fusion) for further functional experiments. Since miR-125b-5p expression levels were very low in these lines, and there is a lack of a protein product to use in measuring knockdown efficiency, only overexpression experiments were performed. miR-125b was overexpressed in these lines using lentiviral based miRNA plasmids (Fig. 5B). MiR-125b overexpression in Res186 (~3 fold), Res259 (~6 fold) and BT66 (~7 fold) uniformly resulted in decreased cell growth (Fig. 5C,D, Supplementary Fig. 3). Differences in miR-125b expression after transfection were likely related to genetic and phenotypic differences between the cells since they are PLGG-derived but with differences in genetic composition.

**Figure 6:**
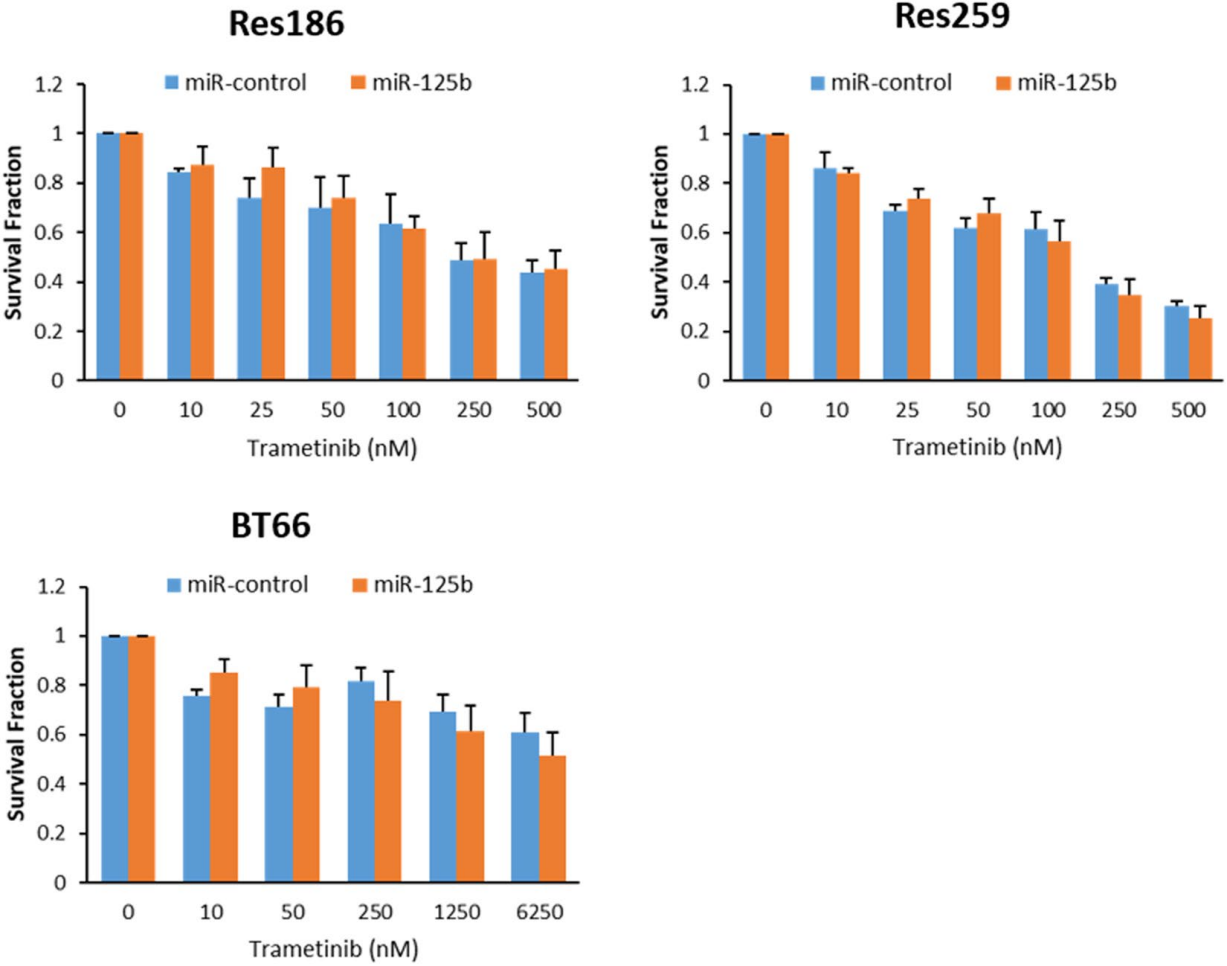
Treatment with the MEK-inhibitor Trametinib has no effect on cell growth in cells with miR-125b overexpression. Evaluation of cell growth after treatment with the MEK-inhibitor Trametinib in BT66, Res186, and Res259 cells overexpressing miR-125b. All data were normalized with DMSO control. The data represents the average of at least 3 biologic replicates.

Since MAPK is activated at the genetic level in almost all PLGG^6^, MEK inhibitors have been used in preclinical and clinical trials for the treatment of this disease. Treatment with the MEK inhibitor trametinib had no significant effect on growth in cells with miR-125b overexpression when compared with control (Fig. 6).

### miR-125b overexpression leads to senescence in BRAF-KIAA1549 PA cells and apoptosis in pediatric low grade glioma lines (PLGG)

**Figure 7:**
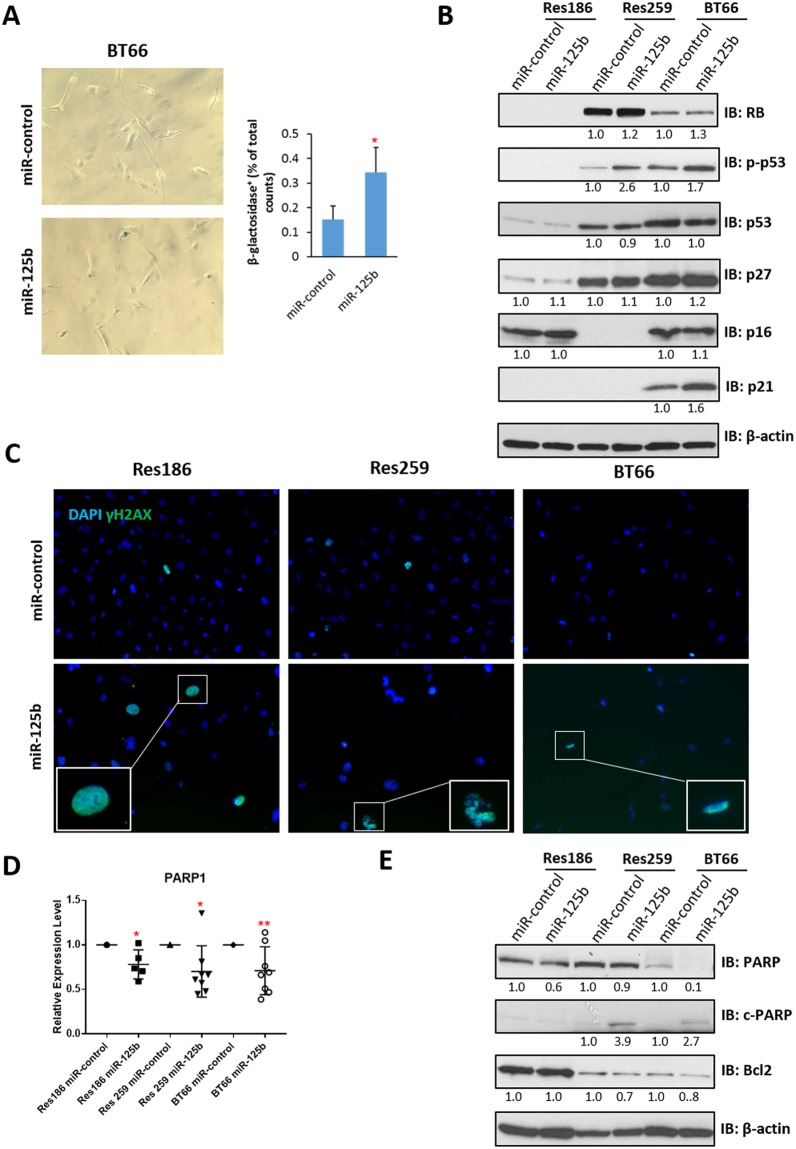
Overexpression of miR-125b induces senescence and apoptosis. (**A**) Representative photomicrographs (200x) of β-galactosidase staining on BT66 cells infected with miR-control or miR-125b, quantification of staining is at right. (*p < 0.05 compare to miR-control). (**B**) Western blot of senescence proteins p16, p21, p27, p53 and RB. β-actin was used as loading control. Protein levels were quantified and normalized to miR-control. (**C**) Representative photomicrographs (200x) of γ-H2AX shows increasing of γ-H2AX positive cells with miR-12b overexpression in all cell lines tested. (**D**) qRT-PCR of PARP1 expression in Res186, Res259 and BT66 (*BRAF* fusion+) cells infected with miR-control or miR-125b. All data were normalized to miR-control for each cell lines. (*p < 0.05, **p < 0.01). (**E**) Western blot of apoptotic protein PARP and the anti-apoptotic protein Bcl2. β-actin was used as loading control. Protein levels were quantified and normalized to miR-control (uncropped Western blots presented as Supplementary Fig. 4). Results represent the average of at least three biological replicates and data were analyzed with a two-tailed Student’s t-test.

Prior melanoma research has demonstrated that miR-125b induces cell senescence^28^. Given the high frequency of *BRAF* mutations in melanoma and PLGG, we hypothesized that senescence may develop in PLGG with *BRAF* alterations. Indeed, overexpression of miR-125b led to induction of acidic β-galactosidase activity in the BT66 cell line (Fig. 7A), but not in Res186 and Res259 (Supplementary Fig. 4). Next, testing for the levels and activation of proteins associated with senescence pathways was performed. The RB1/p16 senescence pathway is dysfunctional in Res186 (with lack of RB1) and Res 259 (with lack of p16). BT66 demonstrated an increase in the senescence marker p21 while p27 was minimally increased (Fig. 7B, Supplementary Fig. 5**)**. BT66 and Res259 had increased levels of p-p53, consistent with activation of the pathway (Fig. 7B). Absence of p53 in Res186 is expected given that it has a *TP53* mutation. The results suggest that senescence after miR-125b overexpression is mediated through TP53/p21 rather than RB1/p16, which is concordant with data published by Nyholm *et al*. in melanoma cells which lack p16^28^.

Given the decrease in cell growth noted after miR-125b we next tested whether apoptosis was a factor. Prior reports have found “panstaining” with γ-H2AX identifies apoptotic cells^35^, so we stained cells that overexpressed miR-125b or miR-125b control using γ-H2AX immunofluorescence. An increased number of γ-H2AX positive cells with a diffuse pattern of staining were present in Res186, Res259 and BT66 after miR-125b overexpression consistent with apoptosis (Fig. 7C). Apoptosis was noted in all three cells lines, including cells with mutant and functional p53. This suggests that apoptosis after miR-125b overexpression in these cells is at least in part p53 independent.

Next, we looked for expression levels of known miR-125b targets, focusing on genes involved in apoptosis. miR-125b overexpression resulted in variable downregulation of these targets at the mRNA level in some but not all the cell lines, including *BBC3* (Res259 and BT66) and *BMF* (Res 259) (Supplementary Fig. 6). The DNA repair enzyme Poly (ADP-ribose) polymerase 1 (PARP1) is also a predicted miR-125b target that was downregulated at the mRNA (20–30%) (Fig. 7D) and protein levels (Fig. 7E, Supplementary Fig. 5) in Res186, Res259 and BT66 overexpressing miR-125b. Static levels of cleaved PARP, a result of apoptosis, were increased in Res259 and BT66 (Fig. 7E). A small increase in cleaved PARP was also identified in Res186 but only when cultured longer (>1 week).

### miR-125b overexpression decreases cell invasion

**Figure 8:**
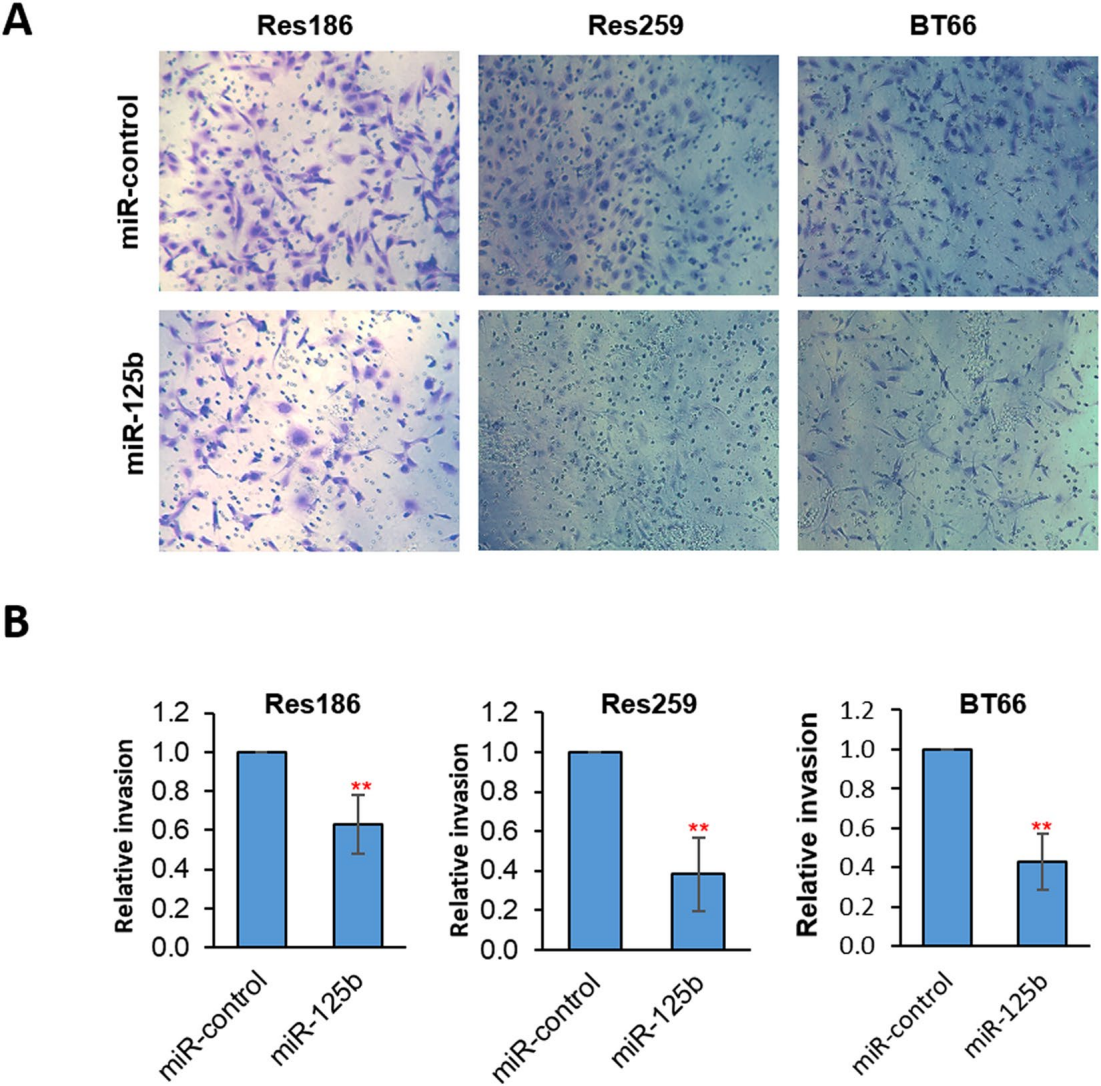
Overexpression of miR-125b decreases cell invasion. Transwell invasion assay of Res186, Res259 and BT66 (*BRAF* fusion+) cells infected with miR-control or miR-125b, migrated cells were photographed (200x) by inverted light microscopy (**A**) or quantitated with Cell-Titer blue (**B**). All data were normalized to miR-control. (**p < 0.01). Results represent the average of at least three biological replicates and data were analyzed with a two-tailed Student’s t-test.

We tested whether miR-125b overexpression affects other glioma cell properties in addition to cell growth. mir-125b overexpression resulted in decreased invasion in Res186, Res259 and BT66 using the transwell assay (Fig. 8).

## Discussion

In the current study, miR-125 members emerged as a microRNA group downregulated in low grade, predominantly pediatric, and glioneuronal tumors compared to diffuse astrocytomas and non-neoplastic brain. The precise reason for this finding is unclear, but our results showed a non-significant trend for lower miR-125b expression in tumors with *BRAF* alterations, with the caveat that only *BRAF* p.V600E and *BRAF* duplications were tested and several other rarer alterations may also affect BRAF and other components of the MAPK pathway in PLGG^6^. When looking at miR-125b levels in pediatric glioma-derived cell lines, we did find variable expression in all cell lines tested, but in general the levels were lower in glioma lines, low and high grade, than in neural stem cell and astrocyte cultures. This raises the possibility that interactions with the microenvironment *in vivo*, may also play a role in miR-125b levels, as it has been reported for other microRNAs in cancer^36^. Overexpression of miR-125b resulted in apoptosis in all three cell lines tested. Senescence, however, was identified only in BT66, a line derived from a PA with *BRAF-KIAA1549* fusion, but not in Res186 and Res259. Our data suggests that miR-125b downregulation may play a role in preventing senescence in tumors that depend on BRAF for cell growth. Known and predicted targets of miR-125b reported in prior studies of cancers characterized by *BRAF* alterations include MLK3, KLF13, CXCL11, and FOXA1. In our study, we found variable levels of miR-125b targets at the mRNA level. The DNA repair enzyme PARP1, however, was a predicted target of miR-125b that was downregulated at the mRNA and protein levels in miR-125b overexpressing cells, particularly BT66. The detection of PARP cleavage products is a reflection of apoptosis, and these were present as well in our studies to various extents in all cell lines tested. Prior studies in ovarian cancer have shown that siRNA PARP knockdowns lead to decreased PARP levels, increased cleaved PARP and apoptosis^37^. Of interest, combination of PARP and MEK inhibitors have been attempted in RAS activated cancers^38^ and may represent a therapeutic opportunity for PLGG. Our observations of miR-125b dysregulation have translational implications, since decreased serum miR-125b has been reported in glioma patients, supporting its potential utility as a biomarker^39^, and efforts to combine miR-125b with chemotherapy and targeted therapies are being explored^40^. We did not see an additive effect of MEK inhibition with trametinib, which is being increasingly applied to tumors with MAPK pathway activation, on cell growth in cells with miR-125b knockdown. Whether this lack of additional effect was a feature of this specific inhibitor or related to a more broad mechanism of MEK inhibitor remains unclear. However, our data suggests that the study of future combination therapies including anti-miR-125b as a strategy may require approaches with other pathway inhibitors and/or chemotherapeutic agents.

There are some limitations regarding the cell lines used for this study. Res186 and Res259 lack known *BRAF* mutations. BT66 maintains a *BRAF* fusion, but was engineered to bypass senescence through the SV40 large T antigen, which is not a feature of PA in general^41^. These efforts represent important attempts to study the biology of PLGG, given that cell lines and models of this disease are relatively unavailable, given the limitations posed by oncogene-induced senescence as mentioned above^29^.

In summary, we identified miR-125b as a biomarker that is under-expressed in PLGG subsets. Overexpression resulted in decreased cell growth, as well as increased apoptosis and senescence in cells containing activated BRAF. Further studies should clarify the specific mechanism of action for this key microRNA and its possible applicability in the management of PLGG patients.

## Materials and Methods

### Patients and Samples

Tumors for *in situ* hybridization were studied using two tissue microarrays, which are constructed using four cores (0.6 mm diameter). A total of 125 patients (median age 17 years, range 1–75) with low grade glioma and glioneuronal tumors (defined as WHO grade I or II) including pilocytic astrocytomas (n = 43), diffuse astrocytoma (n = 24), low grade glioneuronal tumor NOS (n = 3), low grade astrocytoma NOS (n = 8), pleomorphic xanthoastrocytoma (n = 8), angiocentric glioma (n = 4), ganglioglioma (n = 13), dysembryoplastic neuroepithelial tumor (n = 4), and subependymal giant cell astrocytoma (n = 2). Given the rarity of these tumors and the need to broadly represent all the major categories of low grade glial and glioneuronal neoplasms in these tissue microarrays, in addition to tumors developing in pediatric patients (defined as 18 years of age and younger prior to pathologic diagnosis) (n = 66), tumors with the same pathologic characteristics developing in adults (n = 59) were also included in these TMAs. Whole slides were obtained from 3 non-neoplastic seizure brain cases (ages 32, 4 and 2 years) and autopsy samples from a 51 year-old adult, and two fetuses (35 and 25 weeks).

*BRAF* duplication was evaluated using FISH^42^ and *BRAF* p.V600E was evaluated using sequencing^43^ or immunohistochemistry. Immunohistochemistry was performed using an antibody specific for BRAF p.V600E mutant protein (VE1, Ventana, prediluted). All experiments were followed under approval of the Institutional Review Board (IRB) at Johns Hopkins (IRB study number NA_00015113). All research was performed in accordance with all relevant ethical and institutional guidelines/regulations, and the data contains no patient identifiers. The analysis of samples was retrospective and a waiver of consent for the use of patient tissue was applied using all IRB guidelines.

### microRNA profiling data

MicroRNA expression data was obtained from two separate experiments obtained with different platforms. First, global microRNA profiles from 43 PA and 5 non-neoplastic brain control frozen samples were obtained with the Agilent Human miRNA Microarray V3 platform in a previously published study^14^. Fold change was calculated as previously reported^14^, comparing expression in tumor and non-neoplastic brain, after miRNA signal intensities from GeneView files were subjected to quantile normalization using GeneSpring GX, version 11, according to the standard software recommendations (Agilent Technologies). Second, global microRNA profiles from 41 glial/glioneuronal tumors and 4 non-neoplastic brain FFPE samples were obtained using the Nanostring digital counting system in another previously published study^13^. Demographics for individual cases utilized in these studies are outlined in the prior publications.

### Chromogenic *in situ* hybridization studies

For microRNA *in situ* hybridization experiments, Ex miRCURY LNA™ Detection probes, 5′-DIG and 3′-DIG labeled (Exiquon, Woburn, MA), were used. The target probes recognized miR-125b and had the following sequence: 5′-TCACAAGTTAGGGTCTCAGGGA-3′. Positive controls to assess the effectiveness of the reaction included probes recognizing U6 and miR-124, a known brain enriched micrRNA. A probe composed of the scrambled sequence of the miR target was used as a negative control. The FFPE optimization kit was used per manufacturer recommendations. In brief, deparaffination was started by placing slides flat on a warm surface for 1 hour at 60 °C, followed by immersions in xylene X3 for 5 minutes each, and serial passage in ethanol at 100%, 95% and 70%, and PBS. This was followed by incubation with Proteinase-K for 20 minutes at 37 °C. Slides were placed PBS and dehydrated using serial ethanol washes (70%, 95%, 100%). Hybridization mix, with separate slides for target and control probes, was applied and incubated for 1 hour at 55 °C in a hybridizer (DAKO, Ft. Collins, CO). Slides were washed in serial SSC buffers at concentrations of 5x, 1x, and 0.2x. Then, slides were incubated with blocking solution for 15 minutes, followed by application of anti-DIG reagent for 60 minutes (with the reagent replenished after 30 minutes). Slides were washed in PBS-T and incubated with AP-substrate for 2 hours at 30 °C, with fresh solution replenished after 1 hour. Slides were incubated at room temperature in KTBT solution for 5 minutes x2, washed with water and counterstained with Nuclear Fast Red™, rinsed, serially dehydrated with Ethanol (70%, 95%, 100%), and coverslipped using mounting media for light microscopy evaluation. Strength of hybridization was scored using a 4 tiered scale semiquantifiable scale, ranging from 3 (strong, similar to brain), 2 (moderate), 1 (weak), 0 (negative), similar to standard evaluations of cytoplasmic immunohistochemical stains as outline in our prior work (REF). Scoring was performed by a neuropathologist (FJR) blinded to demographic characteristics and pathologic diagnosis, using an Olympus BX43 microscope with a 40X objective. Additional figures were taken using 10X and 20X objectives.

### Cell culture

Pediatric glioma cell lines Res186, Res259 (PLGG derived lines lacking *BRAF* alterations) and UW479 were kindly provided by Dr. Chris Jones (Institute of Cancer Research, Sutton, UK)^44^, and maintained in Dulbecco modified Eagle medium/ F12 Ham medium supplemented with 10% heat-inactivated fetal bovine serum (ThermoFisher Scientific). BT66 originated from a pilocytic astrocytoma containing a *BRAF-KIAA1549* fusion. These cells contain doxycycline-inducible expression of Simian Vacuolating Virus 40 Large T Antigen (SV40-TAg) and stable expressed human Telomerase reverse transcriptase (TERT). BT66 cells were kindly provided by Dr. Till Milde (German Cancer Research Center, Heidelberg, Germany) and maintained in astrocyte growth medium (Lonza) with 1% doxycycline (MilliporeSigma)^41^, cells around passage 38–50 were used for all experiments. TrypLE Express was used for cell dissociation (ThermoFisher Scientific). Pediatric glioblastoma cell line KNS-42 was obtained from the Japan Cancer Research Resources cell bank; CHLA-200 and SJ-GBM2 were obtained from the Children’s Oncology Group (COG) Cell Culture and Xenograft Repository. JHH-CRC1 was derived from a pediatric patient with NF1 associated pilocytic astrocytoma (unpublished data). Human astrocytes were purchased from ScienCell Research Laboratories. Human neural stem cells (hNSC) were kindly provided by Dr. Eric Raabe (Johns Hopkins University, Baltimore, USA)^45^. All cells were cultured in a humidified 37 °C incubator with 5% CO_2_. Cell lines were routinely tested for mycoplasma and maintained in the culture for less than 2 months after thawed from liquid nitrogen. Short tandem repeat (STR) identity testing was performed for Res186, Res259 and BT66 on January 2018.

### miRNA overexpression

Lentiviral based miRNA plasmids were obtained from Biosettia, including expression plasmids pLV-hsa-miR-125b-1 (miR-125b) and pLV-miR-control (miR-control). To produce lentiviruses, 293T cells were transfected with miRNA plasmid and VSVG packaging plasmids mixture using Lipofectamine 2000 (ThermoFisher Scientific). Lentiviral supernatants were collected at 48 h post transfection, passed through a 0.45 micron filter then kept as frozen aliquots at −80 °C until needed. Res186, Res259 and BT66 cells were infected with virus in the presence of polybrene (5 µg/ml, MilliporeSigma).

### Cell growth and cell survival assay

The CellTiter-Blue cell viability assay kit (Promega) was used to count viable cells. 72 hours after virus infection, cells were seeded in 96 well plates at a density of 1000 cells per well in growth media. Twenty microliters of CellTiter-Blue reagent was added to 100 µl medium and incubated for 1 hour at 37 °C incubator. Fluorescence (560 nmEx/590 nmEm) was continuously measured for 5 days using TECAN plate reader. For drug treatment, cells were cultured in the growth media with 2% heat inactived FBS with different doses of trametinib (Skellchem) for 5 days, DMSO was used as vehicle control. Relative cell numbers were converted with standard curve of each cell line.

### Quantitative real-time polymerase chain reaction (qRT-PCR)

Primers for miRNA were designed by using miRprimer software^46^, sequences can be found in Supplementary Table 1, small RNA RNU48 was used as the endogenous control. Primer sequences for mRNA target genes were listed in Supplementary Table 2, *HPRT1* was used as the endogenous control. Total RNA was isolated from cultured cells using miRNeasy mini kit (QIAGEN), reverse transcription of miRNA was performed according to Peter K Bush’s protocol^46^, and cDNA for target genes were produced using QuantiTect reverse transcription kit (QIAGEN). qRT-PCR was performed using PowerUp SYBR Green Master Mix (ThermoFisher Scientific) according to the manufacturer’s protocol. The relative fold changing was calculated using the formula 2^ΔCT^.

### Bromodeoxyuridine (BrdU) incorporation assay

72 hours after virus infection, cells were incubated with 10 µM BrdU (MilloporeSigma) for 2 hours, dissociated and fixed with 70% ethanol overnight at 4 °C. Cells were washed with PBS and incubated with 2 N HC for 30 minutes at 37 °C. After three times of PBS washing, cells were permeabilized with 0.1% Triton and stained with BrdU-PE (eBioscience) for 30 minutes at room temperature, analyzed with Muse flow cytometer (MilloporeSigma).

### *In vitro* invasion assay

Cell invasion assay was analyzed using a 24 well system with growth factor reduced Matrigel coated transwell inserts containing 8 µM pore membrane (Fisher Scientific). 72 hours after virus infection, 1 × 10^5^ cells in 250 µl of DMEM/F12 were added on top of each membrane and fill bottom with 750 µl of complete growth medium. After 22 hours, migrated cells from membrane were fixed and stained with 0.05% crystal violet in 50% methanol or dissociated by using TrypLE Express (ThermoFisher Scientific), relative cell numbers were measured by using Cell-Titer blue reagent (Promega) and converted with standard curve of each cell line.

### Western blotting

Cells were lysed in RIPA lysis buffer supplemented with protease inhibitors (MilliporeSigma). Protein extracts were loaded on 4–12% Bis-Tris gel. Antibodies used for western blot were: Bcl2 (#2870, 1:1000, Cell Signaling Technology), p16 (sc-56330,1:200, Santa Cruz), p21 (#2947, 1:1000, Cell Signaling Technology), p27 (sc-1641,1:500, Santa Cruz), p53 (#9282, 1:1000, Cell Signaling Technology), Phospho-p53 (Ser15) (#9286, 1:1000, Cell Signaling Technology), PARP (#9542, 1:1000, Cell Signaling Technology), Cleaved PARP (Asp214) (#5625, 1:1000, Cell Signaling Technology), RB (#9309, 1:2000, Cell Signaling Technology), β-actin (sc-47778, 1:5000, Santa Cruz). The appropriate HRP-conjugated secondary antibodies (1:5000, Cell Signaling Technology) was used and blots were revealed by chemiluminescence (ECL, PerkinElmer). For Phospho-p53 and p53 western blots, membrane was stripped with restore western blot stripping buffer (ThermoFisher Scientific) and re probed. Densitometry analysis was performed using ImageJ software.

### Senescence Assay

β-galactosidase staining was performed as per the manufacturer’s instructions (#9680, Cell Signaling Technology). Cells were infected with miR-125b or miR-control virus, 72 hours later 50000 cells were plated on 12-well plates. After 48 hoursculture, cells were fixed and stained for overnight. Cells were photographed by inverted light microscopy with 20x objective.

### Immunofluorescence staining for γ-H2AX

For immunofluorescence staining, cells were cultured on the glass coverslips and fixed with 4% paraformaldehyde for 15 min. After three times washing with PBS, cells were incubated for 45 min in blocking buffer containing PBS, 1% bovine serum albumin, 0.1% Triton-X-100 and 5% donkey serum, then incubated overnight at 4 °C with anti-γ-H2AX antibody (#2870, 1:200, Cell Signaling Technology). Cells were washed three times with PBS and incubated for 1 hour with Fluorescein (FITC) conjugated Donkey Anti-rabbit IgG (H + L) (711-095-152, 1:100, Jackson ImmunoResearch Laboratories). DAPI (MilliporeSigma) solution was applied to stain nuclei. Cells were mounted with Prolong Gold anti-fade mounting media (ThermoFisher Scientific) and visualized by Motic AE31 inverted microscopy with a 20x objective.

### Statistical Analysis

Means and ranges were used to describe the *in situ* hybridization scores as appropriate. Statistical tests were performed using Associations with clinicopathologic variables was assessed with contingency tables and the Chi Square test. Continuous variables were compared with the Wilcoxon/Kruskal-Wallis Test. Statistical analyses were performed using JMP v10 software (SAS Institute). Analyses were 2-sided with p < 0.05 considered statistically significant. For cell culture and functional assays, data were presented as mean ± s.d, with p < 0.05 considered statistically significant. All *in vitro* experiments described in the above sections (including gene expression data, cell proliferation data, H2Ax assays, senescence assays), were performed in at least three biological replicates and data were analyzed with a two-tailed Student’s t-test.

## Electronic supplementary material

Supplementary Information
