# Supplementary material for: MicroRNA (miR) 125b regulates cell growth and invasion in pediatric low grade glioma
Source: Sci Rep. 2018 Aug 21;8:12506. doi: 10.1038/s41598-018-30942-4 (PMC6104092; doi:10.1038/s41598-018-30942-4)
Supplement: Supplementary file 1 — Supplementary Information [file 41598_2018_30942_MOESM1_ESM.pdf]

MicroRNA (miR) 125b regulates cell growth and invasion in pediatric low grade glioma

Ming Yuan[1*], Ana Cristina A.L. Da Silva[1,2]*, Antje Arnold[1], Laurence Okeke[1], Heather Ames[1], Lina S. Correa-Cerro[1], M. Adelita Vizcaino[1,3], Cheng-Ying Ho[4], Charles G. Eberhart[1,5], Fausto J. Rodriguez[1,5]

Supplementary figure 1. miR-125b expression in adult brain tissue. miR-125b was expressed in mature neurons, but weakly expressed to absent in white matter. U6 served as positive control and scramble sequences as negative control.

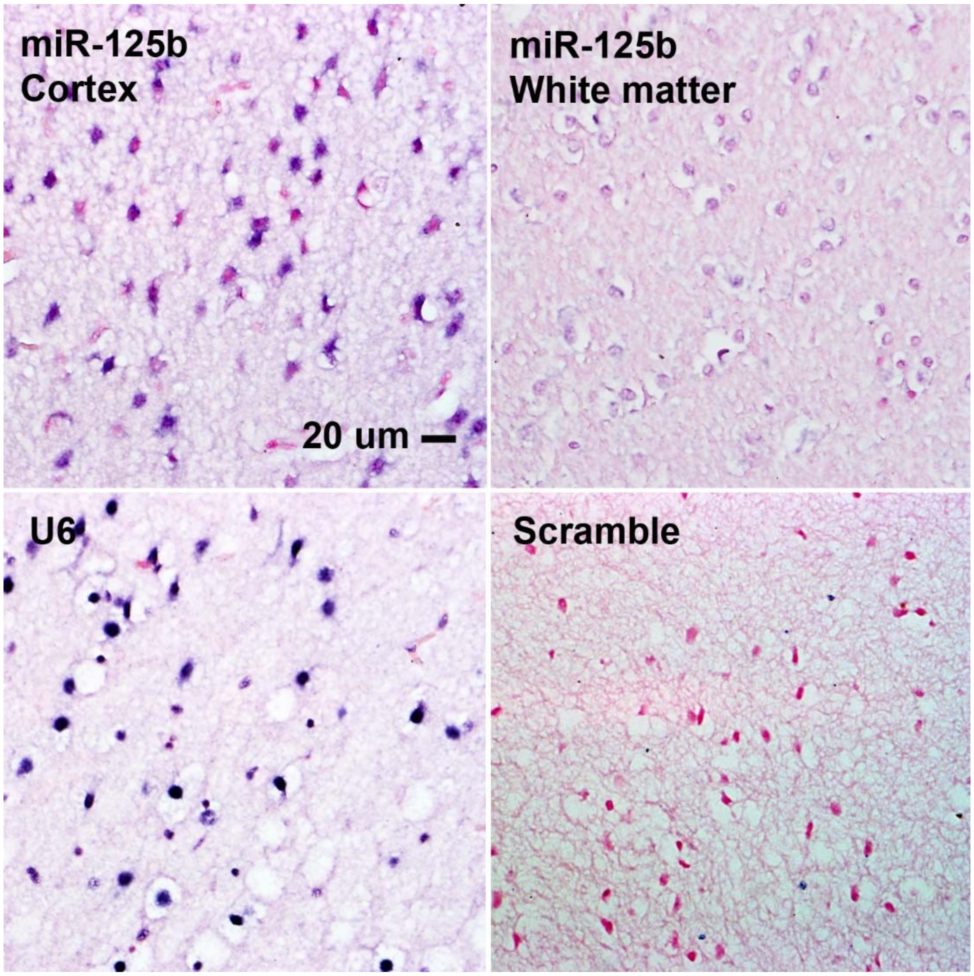

Supplementary figure 2. MiR-125b-1-3p and miR-125b-2-3p expression in pediatric cell lines analyzed through qRT-PCR. All data were normalized with RNU48.

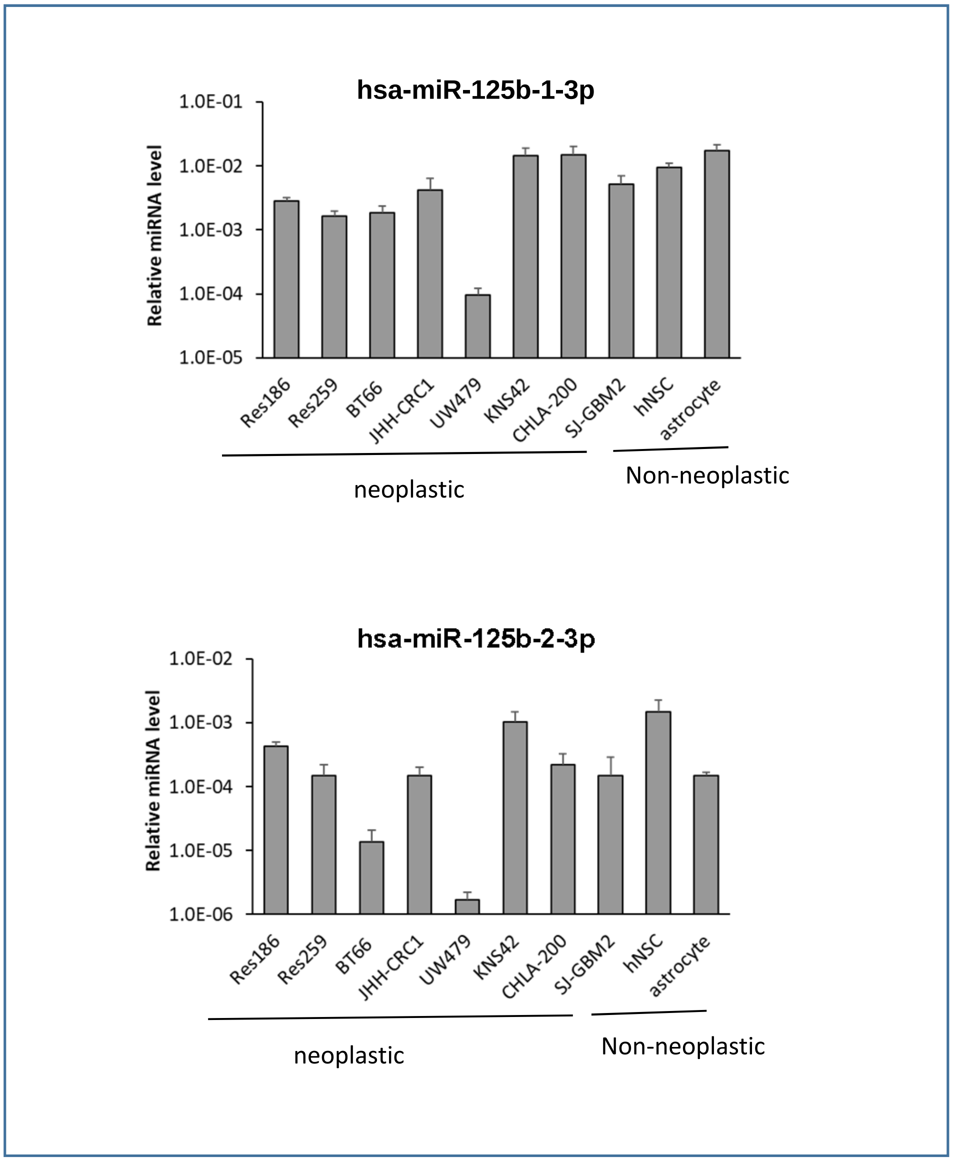

Supplementary figure 3. Representative photomicrographs (200x) of Res186, Res259 and BT66 cells . 1×10^6 cells were infected with miR-control or miR-125b, 72 hours later, cells were photographed or counted. (* $p<0.05$, ** $p<0.01$ compared to miR-control).

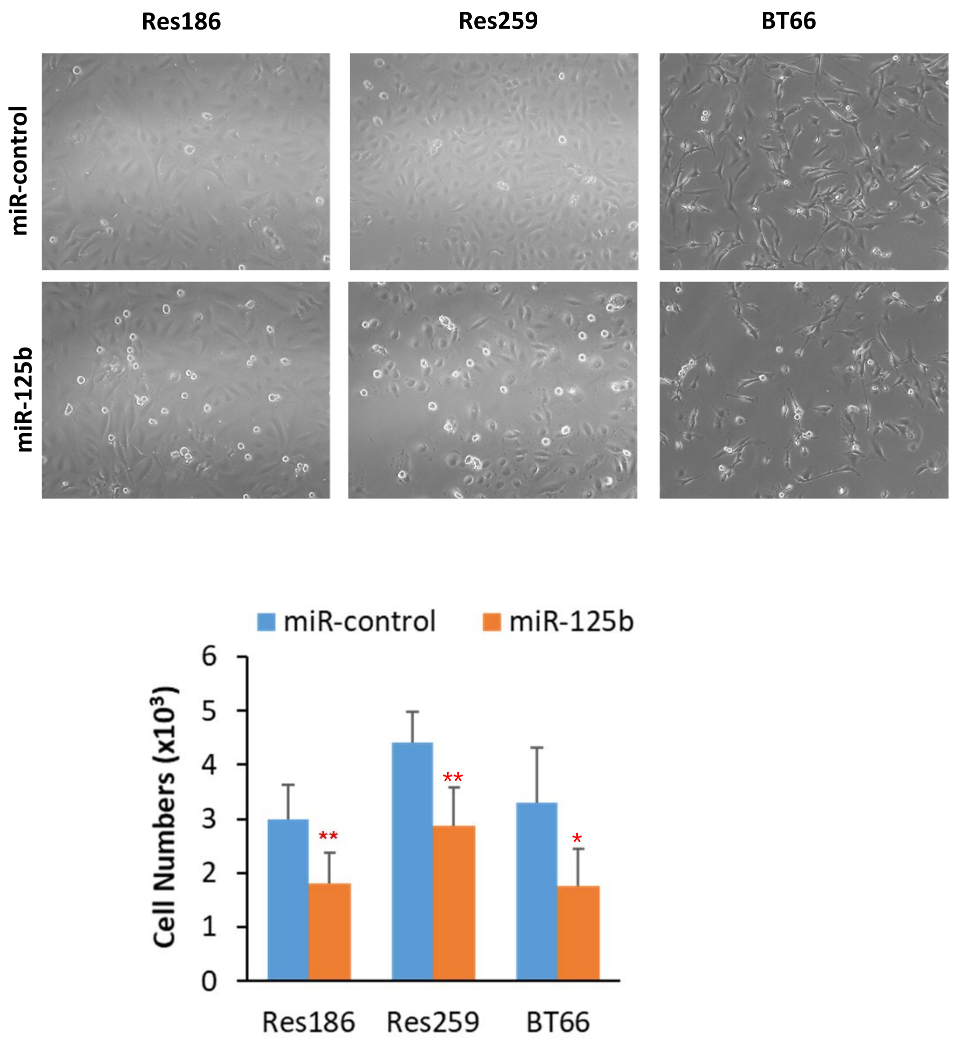

Supplementary Figure 4. Representative photomicrographs (200x) of β-galactosidase staining on Res186 and Res259 cells.

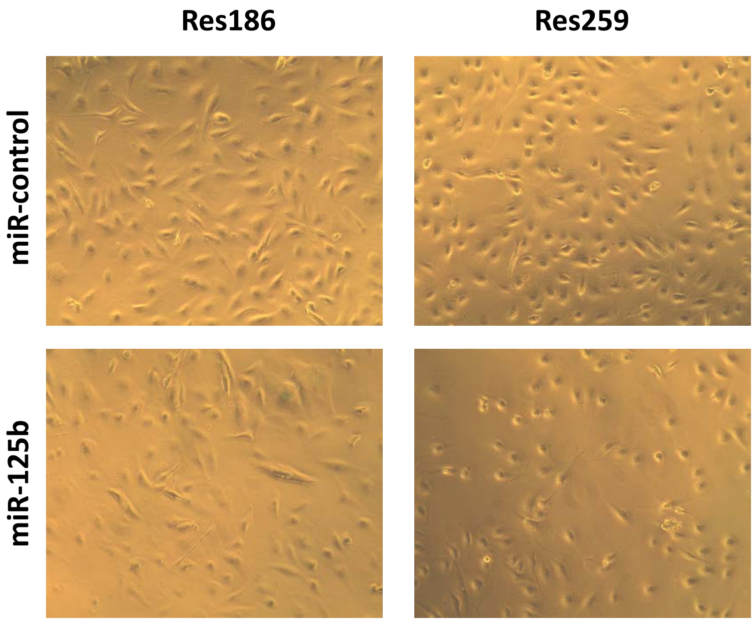

Supplementary Figure 5. Western blots after overexpression of miR-125b in PLGG derived cell lines Res 186, Res 259, BT66.

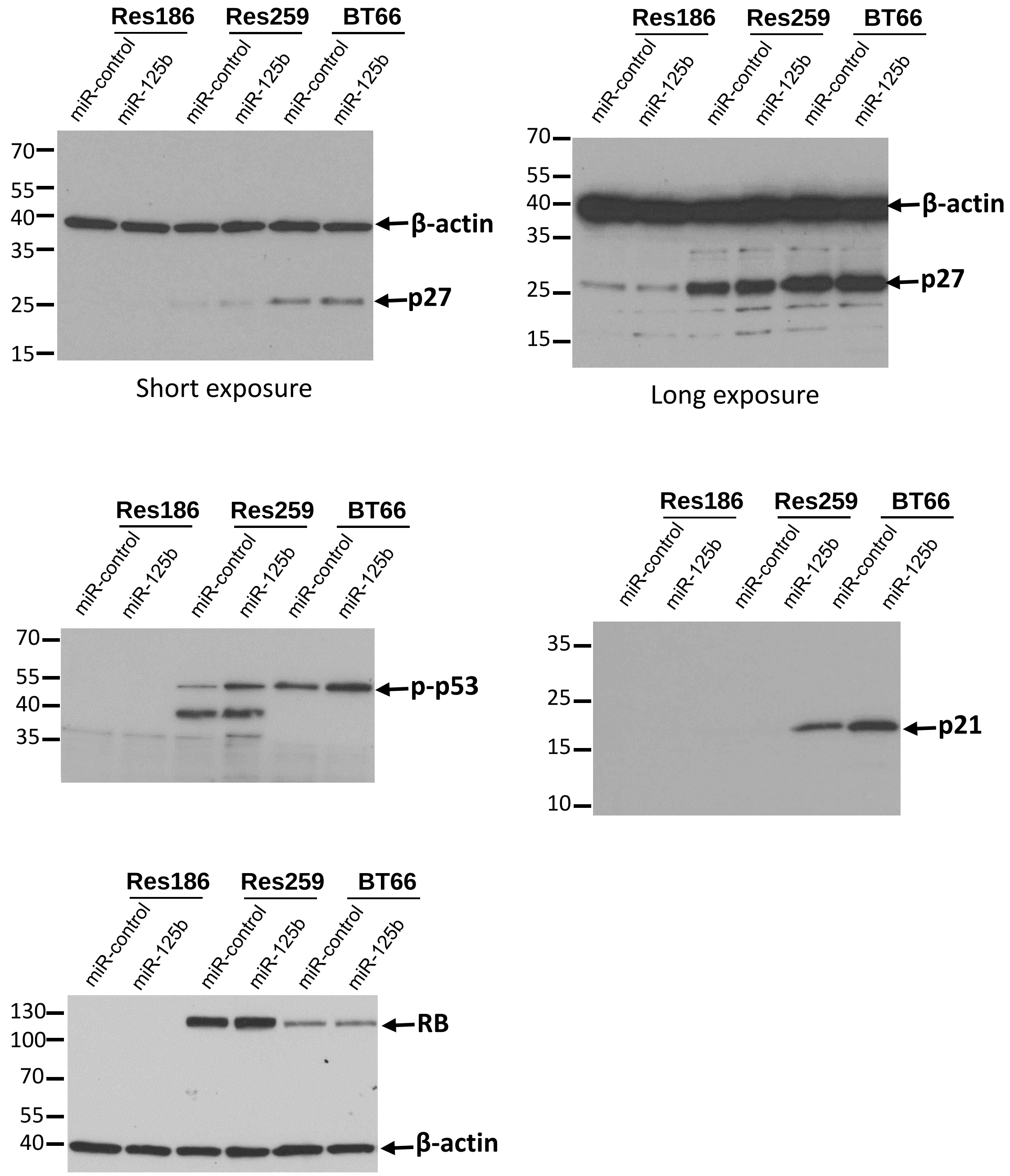

Supplementary Figure 5 (continued)

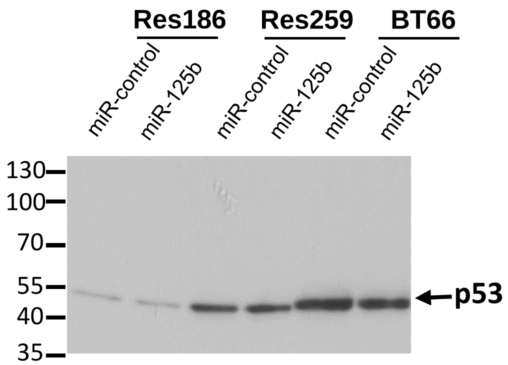

Short exposure

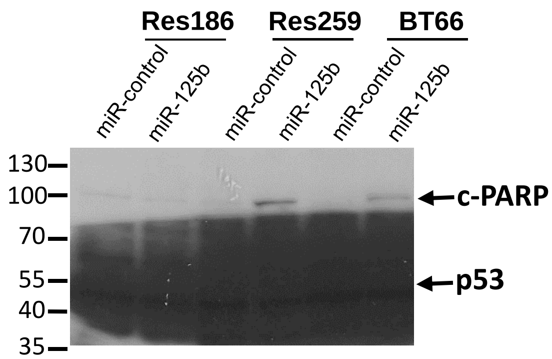

Long exposure

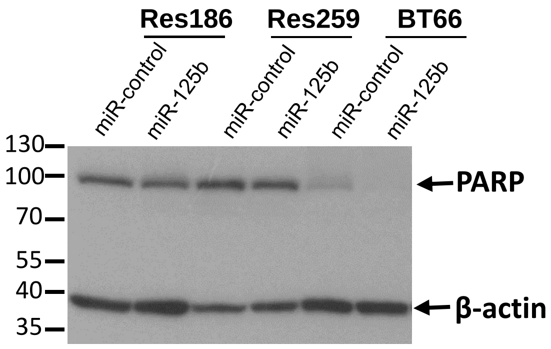

Short exposure

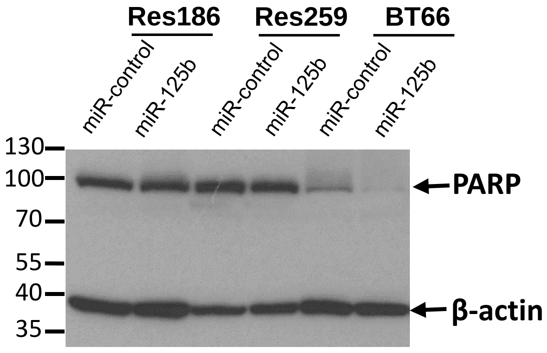

Long exposure

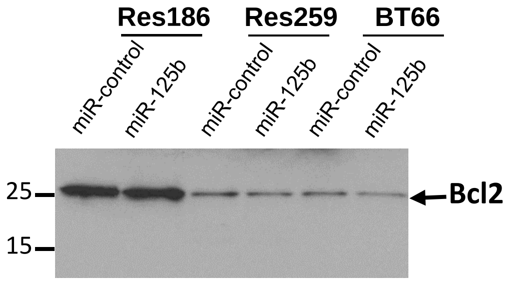

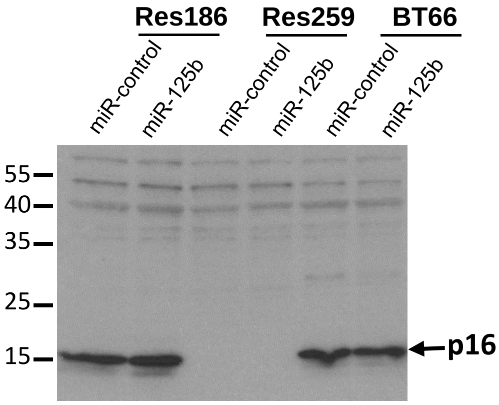

Supplementary Figure 6. Effect of miR-125b overexpression on predicted mRNA targets invoved in apoptosis. qRT-PCR of miR-125b potential target in Res186, Res259 and BT66 cells infected with miR-control or miR-125b. All data were normalized to HPRT1. (* $p<0.05$, ** $p<0.01$). Each point represent an independent experiment.

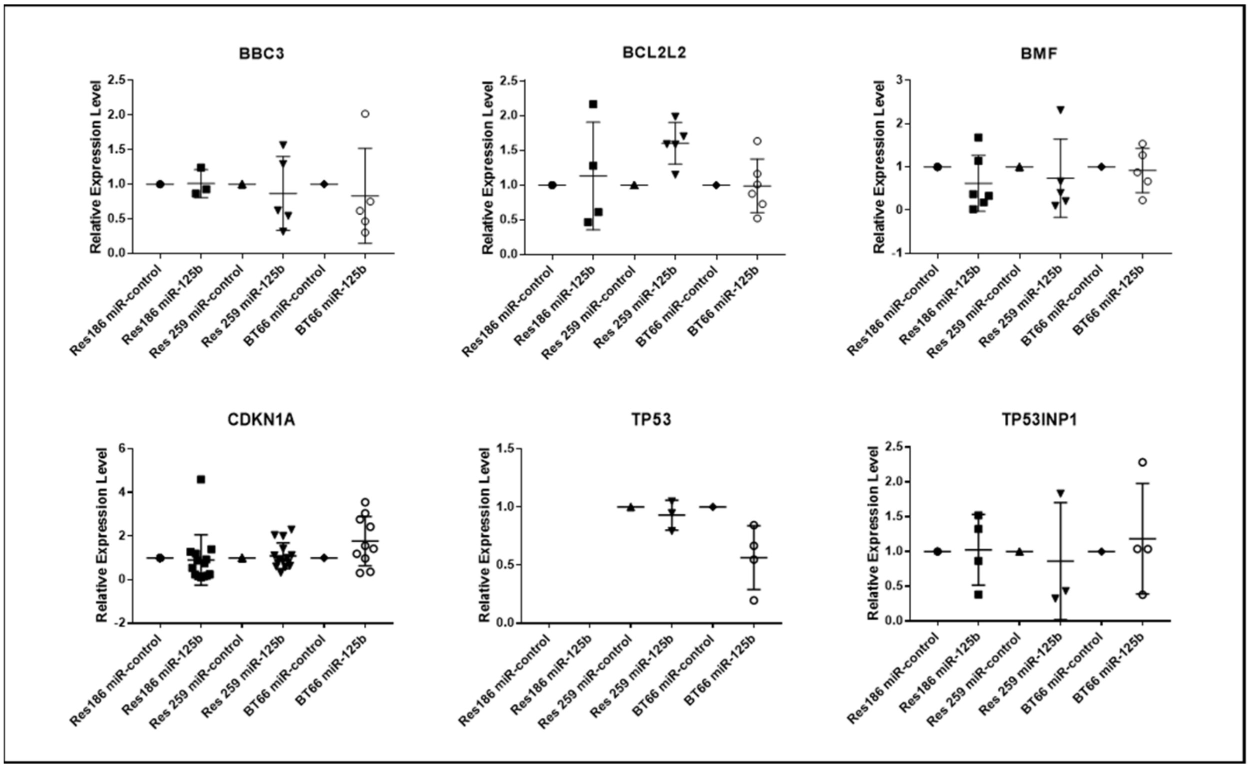

Table 1. qRT-PCR primers for miRNA

miRNA name	forwarder primer	reverse primer
hsa-miR-125b-5p	5'-CAGTCCCTGAGACCCTAAC-3'	5'-GTCCAGTTTTTTTTTTTTTTTCACAAG-3'
RNU48	5'-AGTGATGATGACCCCAGGTAACTC-3'	5'-CTGCGGTGATGGCATCAG-3'

Table 2. qRT-PCR primers for miRNA target genes

gene name	forwarder primer	reverse primer
BBC3	5'- GACGACCTCAACGCACAGTA -3'	5'- GTAAGGGCAGGAGTCCCAT -3'
BCL2L2	5'- GGACAAGTGCAGGAGTGGAT -3'	5'- CGTCCCCGTATAGAGCTGTG -3'
BMF	5'- TTGTGGGGTGACTGAGGAAC -3'	5'- TTCAAAGCAAGGTTGTGCAG -3'
CDKN1A	5'- AGTCAGTTCCTTGTGGAGCC -3'	5'- CATGGGTTCTGACGGACAT -3'
PARP1	5'- GATGGGTTCTCTGAGCTTCG -3'	5'- TCTGCCTTGCTACCAATTCC -3'
TP53	5'- GCTTTCCACGACGGTGAC -3'	5'- GCTCGACGCTAGGATCTGAC -3'
TP53INP1	5'- CTGTGCATAACTCCTGCCCT -3'	5'- TATGCTGCCCCATTTCATTT -3'
HPRT1	5'- GTTATGGCGACCCGCAG -3'	5'- ACCCTTTCCAAATCCTCAGC -3'

Supplementary Table 3: Tissues with increased miR-125b expression levels in reads per million (RPM)

miRNA	hsa-miR-125b-1-3p	hsa-miR-125b-2-3p	hsa-miR-125b-5p
Prefrontal Cortex	278	2,029	22,005
Frontal Brain	388	1305	16694
Cerebellum	8	553	9194
Forebrain	26	203	176
Midbrain	12	1544	1130
Retina	59	353	4149
RPE	267	138	67150
Epidermis	0	713	14954
Dermis	94	388	35953
Skin	67	543	58488
Cervix	34	81	30022
Neural stem cell	24	161	222
Neuron_iPSC	730	621	15844
Astrocytes_JH-01.fastq	1725	607	9300
Derm_Fib_Ad_JH-06.fastq	1142	529	9223
Derm_Fib_Neo_JH-07.fastq	3907	565	12526
Osteoblasts_JH-15.fastq	1966	177	21736
Per_lig_Fib_JH-22.fastq	2823	348	20287
Sk_Mus_Cell_JH-25.fastq	3199	451	15721
JH-03_Chondr.fastq	2161	683	15550
JH-08_Card_fib.fastq	1907	146	14853
Valve interstitial cell	893	100	23430
iPSC-CM	185	37	34972
